# Supplementary material for: Superior long-term patency of no-touch vein graft compared to conventional vein grafts in over 1500 consecutive patients
Source: J Cardiothorac Surg. 2024 Oct 1;19:570. doi: 10.1186/s13019-024-03057-3 (PMC11443723; doi:10.1186/s13019-024-03057-3)
Supplement: Supplementary file 1 — Supplementary Material 1 [file 13019_2024_3057_MOESM1_ESM.docx]

| **Patient characteristics** | **N** |  |
| --- | --- | --- |
| NT to LAD | 70 |  |
| C to LAD | 66 |  |
| NT to DA | 360 |  |
| C to DA | 519 |  |
| NT to MA | 468 |  |
| C to MA | 673 |  |
| NT to RCA | 404 |  |
| C to RCA | 623 |  |

Table S1: Grafts characteristics of the two groups divided by territory of distribution.

LAD: left anterior descending, DA: diagonal artery, MA: marginal artery, RCA: right coronary artery.

Table S2: Patency rates of the arterial grafts.

| **Patient characteristics** | **Patency** |  |
| --- | --- | --- |
| No. of patients | 1291 |  |
| Patency Grafts (%) |  |  |
| All | 86.3 |  |
| LITA | 96.2 |  |

Values are presented as %. LITA: left internal thoracic artery.

Table S3: Patency per patient divided by the type of vein graft

| **Patient characteristics** | **No-touch** | | **Conventional** | | **p-value** |
| --- | --- | --- | --- | --- | --- |
| No. of patients | | 618 | | 825 |  |
| Patient Patency | | 70.7 | | 46.7 | <0.001 |
| Single | | 75.9 | | 47.1 | <0.001 |
| Sequential | | 68.3 | | 46.1 | <0.001 |
